# S100-alarmins, antenatal corticosteroids and the risk of late-onset sepsis in preterm infants: A prospective cohort study

**DOI:** 10.1371/journal.pone.0341544

**Published:** 2026-01-27

**Authors:** Gloria Kessler, Thomas Ulas, Thomas Vogl, Johannes Roth, Fenja Albrecht, Gesine Hansen, Constantin S. von Kaisenberg, Christoph Härtel, Bettina Bohnhorst, Dorothee Viemann, Sabine Pirr

**Affiliations:** 1 Department of Pediatric Pneumology, Allergology and Neonatology, Hannover Medical School, Hannover, Germany; 2 Genomics and Immunoregulation, LIMES-Institute, University of Bonn, Bonn, Germany; 3 Institute of Immunology, University of Münster, Münster, Germany; 4 Cluster of Excellence RESIST (EXC 2155), Hannover Medical School, Hannover, Germany; 5 Department of Obstetrics, Gynecology and Reproductive Medicine, Hannover Medical School, Hannover, Germany; 6 Department of Pediatrics, University Hospital Würzburg, Würzburg, Germany; 7 Centre for Infection Research, University of Würzburg, Würzburg, Germany; Kobe University Graduate School of Medicine School of Medicine, JAPAN

## Abstract

**Objectives:**

Antenatal corticosteroids (aCS) are an important measure improving the outcome of preterm infants. Their influence on late-onset sepsis (LOS) risk remains inconclusive. The alarmin S100A8/A9 protects from LOS by regulating innate immune responses. We examined whether aCS impact on postnatal S100A8/A9 serum-levels and consequently on LOS risk.

**Study design:**

In a prospective birth-cohort study of 162 preterm infants born before 32 gestational weeks, we determined postnatal S100A8/A9 serum-levels in relation to the timing of aCS and LOS incidence.

**Results:**

aCS administration within 7 days before birth decreased LOS incidence in infants born via primary C-section compared to infants not exposed to aCS (5/69 (7.2%) vs. 4/27 (14.8%)). This effect was linked to increased S100A8/A9 levels, with nocturnal aCS administration being most effective. Opposite, S100A8/A9 levels were lower and the LOS incidence higher compared to unexposed infants (7/23 (30.4%) vs. 4/27 (14.8%)) when aCS were administered more than 14 days before delivery.

**Conclusion:**

Our data suggest that aCS administration affects the risk of LOS in preterm infants in dependence of the timing of administration by influencing the infant’s S100A8/A9 levels. This underlines the importance of optimal timing of aCS facing imminent preterm birth.

## Introduction

Corticosteroid administration to mothers before anticipated preterm birth is one of the most important antenatal therapies available to improve newborn outcomes [1,2]. Neonates whose mothers received antenatal corticosteroids (aCS) have a significantly lower risk of respiratory distress syndrome, perinatal and neonatal death, intraventricular hemorrhage and necrotizing enterocolitis [1–3]. A single course of corticosteroids consisting of two doses of 12 mg betamethasone given 24 hours apart is recommended for pregnant women between 24^+0^ and 33^+6^ weeks of gestation, who are at risk of preterm delivery within 7 days [3,4]. They accelerate fetal organ maturation and induce surfactant release [5,6]. However, the molecular mechanisms are not fully understood. The benefit of corticosteroid administration in reducing mortality and morbidity is greatest at one to 7 days after the initial dose [1,7,8].

Inconsistent results have been reported on the effect of aCS on the risk of late-onset sepsis (LOS) in preterm infants. Whereas Amorim et al. showed a reduction in neonatal infections after aCS treatment [9], several other studies could not detect a beneficial effect of aCS on the incidence of LOS [10–12]. Recently, a nationwide cohort study from Taiwan found that children exposed to one course of antenatal corticosteroids were at a significantly increased risk of serious infection during the first 12 months of life regardless of gestational age (GA) at birth [13]. This ambiguity points to unconsidered factors, e.g., the timing of aCS administration, which determine the effect of aCS on the risk of LOS.

We previously demonstrated that the serum level of S100A8/A9 (also known as calprotectin) during the first three days of life is the factor with the highest effect size on the risk of LOS compared to other hitherto identified risk factors [14]. S100A8/A9 is an essential regulator of neonatal immunity protecting from sepsis by preventing exceeding inflammatory responses to microbial challenges [14–17]. S100A8/A9 is rapidly released from myeloid cells upon stress [18] and serum levels are physiologically high after birth [19], particularly when accompanied by labor-related stress [14,17]. Previous murine and adult patient studies demonstrated an upregulation of S100A8/A9 expression by synthetic steroids and glucocorticoids [20–22] suggesting that aCS might influence S100A8/A9 serum levels in preterm newborn infants.

In this prospective cohort study, we demonstrate that timing of aCS is associated with S100A8/A9 levels and LOS incidences in preterm infants. Particularly in infants born by primary cesarean section (C-section), aCS given within one week prior to birth increase S100A8/A9 serum levels and decrease the risk of LOS. Greater time intervals between aCS and birth are linked to lower postnatal S100A8/A9 serum levels and increased risk of LOS.

## Materials and methods

### Study population

In a preterm infant birth cohort, clinical metadata and 304 serum samples from cord blood and peripheral blood on day one to day three of life were prospectively collected from 162 infants born at a GA of 24–31 weeks at the Hannover Medical School from February 2014 to February 2019 (Table 1). Mothers and infants with amnion infection syndrome or early-onset sepsis were excluded from analyses as well as infants with major congenital malformation, inborn errors of metabolism, immunodeficiencies or perinatal asphyxia, and births resulting from maternal trauma. For the collection of clinical metadata children were followed until discharge or death by trained research staff. The administration of aCS to the mothers was determined by the attending senior gynecologist if premature delivery was considered likely within the next 7 days. The time of administration was taken from the nurses’ documentation in the mother’s medical records and documented accordingly.

**Table 1:** Clinical characteristics of the preterm infant birth cohort.

	All (n = 162)	Corticosteroids prior to birth					P value
		Non or incomplete (n = 27)	1 to 7 days (n = 69)	8 to 14 days (n = 28)	15 to 21 days (n = 23)	22 to 28 days (n = 15)	
Gestational age, weeks, mean (SD)	28.6 (2.1)	29.0 (1.7)	28.3 (2.2)	28.4 (2.5)	29.4 (1.7)	28.0 (0.9)	0.0835^a^
Birth weight, g, mean (SD)	1125 (390)	1241 (424)	1091 (390)	1063 (403)	1232 (389)	1029 (236)	0.1482^a^
Birth weight percentile, mean (SD)	40 (25)	47 (28)	40 (25)	34 (22)	37 (21)	43 (29)	0.3183^a^
SGA, n (%)	24 (14.8)	5 (18.5)	11 (15.9)	4 (14.3)	2 (8.7)	2 (13.3)	0.8978^b^
Sex, female, n (%)	64 (39.5)	10 (37.0)	31 (44.9)	12 (42.9)	6 (26.1)	5 (33.3)	0.5543^b^
Cesarean section, n (%)	134 (82.7)	21 (77.8)	55 (79.7)	25 (89.3)	18 (78.3)	15 (100)	0.2678^b^
Primary cesarean section, n (%)	81 (50.0)	12 (44.4)	39 (56.5)	14 (50.0)	12 (52.2)	4 (26.7)	0.3066^b^
Reason for preterm delivery, n(%)							0.2007^a^
Premature labor or PROM	68 (42.0)	13 (48.2)	28 (40.6)	10 (35.7)	10 (43.5)	7 (46.7)	
Pathologic CTG or Doppler	66 (40.7)	9 (33.3)	29 (42.0)	11 (39.3)	11 (47.8)	6 (40.0)	
Abruption of placenta	7 (4.3)	1 (3.7)	1 (1.5)	3 (10.7)	1 (4.3)	1 (6.7)	
Preeclampsia/ HELLP	14 (8.7)	2 (7.4)	8 (11.6)	2 (7.1)	1 (4.3)	1 (6.7)	
Maternal disease	7 (4.3)	2 (7.4)	3 (4.3)	2 (7.1)	0 (0.0)	0 (0.0)	
Sample day of life, mean (SD)	1.2 (1.1)	1.4 (1.0)	1.3 (1.1)	1.0 (1.1)	1.3 (1.1)	1.1 (1.1)	0.3821^a^
LOS, n (%)^d^	21 (13.0)	4 (14.8)	5 (7.2)	4 (14.3)	7 (30.4)	1 (6.7)	0.0683^b^

^a^Kruskal-Wallis Tests with post-hoc Dunn’s multiple comparisons.

^b^Chi^2^ Test

^c^Three infants presented with a grade 4 ANS, two with an incomplete course of antenatal corticosteroid, one with antenatal corticosteroids one day before birth.

^d^Eight (40%) cases of LOS were blood culture proven (one *Escherichia coli*, two *Bacillus cereus*, one CoNS, one *Klebsiella pneumoniae*, two *Enterobacter aerogines*, one *Enterobacter cloacae*).

### Definitions

A complete course of aCS consisted of 2 doses of 12 mg betamethasone administered 24 hours apart [3,23]. All mothers received the same dosage of 12 mg per administration. When birth occurred before the second dose, the course was considered incomplete. Patients were stratified according to the time interval between the first dose of betamethasone and birth in 7-day sections. Infants whose mothers did not receive any or an incomplete course of aCS due to uncontrollable labor or an urgently medically indicated delivery were defined as control group. Infants whose mothers had received more than one complete course of aCS were excluded from the analysis as an influence of the additional aCS course and its timing on the outcome of the infants could not be ruled out, making a clear assignment to the subgroups defined above impossible.

Gestational age (GA) was calculated based on the last menstrual period. If the early ultrasound at 11–13 + 6 weeks of gestation showed a difference of more than seven days in the fetal crown-rump length, dating was performed using ultrasound.

Primary C-section was defined as C-section at the absence of labor.

Neonatal sepsis was defined based on a validated sepsis score implemented by the German nationwide surveillance system for sepsis in very low birth weight infants (National Surveillance System NEO-KISS) [24]. Clinical sepsis was diagnosed in the presence of at least two clinical criteria (temperature >38°C or <36.5°C, tachycardia >200/min, occurrence or increase of hypoxemias, bradycardias or apneas requiring intensification of respiratory support or medication, hemodynamic instability, hyperglycemia >10mmol/l, metabolic acidosis, greyish skin color, and prolonged reperfusion time) or one clinical and at least one laboratory sign (CRP > 20mg/l, IL-6 > 300ng/l, a ratio of immature to total neutrophils of >0.2, white blood cell count <5/nl, and platelet count <100/nl) and antibiotic treatment for a minimum of five days, but no proof of causative agent in the blood culture. Blood-culture proven sepsis was defined as clinical sepsis with pathogen growth in the blood culture. If coagulase negative staphylococci (CoNS) were detected as single pathogen in the blood culture, two separate positive cultures were mandatory to consider CoNS as the sepsis causative agent. Clinical and laboratory signs of infants diagnosed with LOS in our cohort are shown in S1 Table.

Early-onset sepsis was defined as sepsis occurring within the first 72 hours after birth, LOS was defined as sepsis after the first 72 hours of life.

### Measurement of S100A8/A9 concentrations

Serum samples were centrifuged at 3000 × g at room temperature for ten minutes and stored at −80°C. S100A8/A9 was determined by an in-house ELISA as described previously [18].

### Statistical analysis

Data were analyzed anonymously and tested for Gaussian distribution using the Shapiro-Wilk normality test. For subgroup comparisons Chi^2^ tests and Kruskal-Wallis tests with *post-hoc* Dunn’s multiple comparison test and Mann Whitney *U* tests were applied as indicated in the results using GraphPad Prism® (version 9; GraphPad software, San Diego, CA, USA). To analyze the associations between the administration of aCS, S100A8/A9 serum levels and clinical outcomes multiple regression analyses were performed using a generalized logistic model while taking clinical characteristics (GA, mode of delivery (MOD), reason for preterm delivery) of the infant into account using GraphPad Prism®. The association between the timing of aCS administration and S100A8/A9 serum levels on day one to day three of life was assessed by employing a nested model test building generalized linear mixed effect models (R lme4 package [25]) of measured data using a Gamma distribution error model and setting zero values to a pseudocount floor of 1-e-10. Thereby, the model including day of aCS administration, MOD, sex, and birth weight (BW) percentile was contrasted to a model containing MOD, sex, and BW percentile only to test for the independent effect of the timing of aCS administration using a likelihood ratio test. The effect sizes of timing of aCS administration, MOD, sex, and BW percentile on S100A8/A9 serum levels were calculated by employing a generalized linear model and visualized using the R package sjplot (v2.8.3; URL: https://CRAN.R-project.org/package=sjPlot) and function plot-model [26] using default parameters. A *P* value <0.05 was considered statistically significant. Within the figures, lines with stops indicate statistical results of all subgroups within the two stops. Simple lines indicate statistical results between the two subgroups connected by the line.

### Ethics statement

Study approval statement: Protocols were approved by the Institutional Review Board of the Hannover Medical School (no. 6031–2011, no. 6031–2015, Research Obstetrics Biobank no. 1303–2012) and were in accordance with the Helsinki Declaration (1964, amended most recently in 2008) of the World Medical Association.

Consent to participate statement: Written informed consent was obtained from the parents of participating infants.

## Results

### aCS influence the occurrence of LOS

The occurrence of LOS in our cohort of preterm infants (Table 1) was determined in dependence of the time interval between aCS administration and preterm birth.

**Fig 1:**
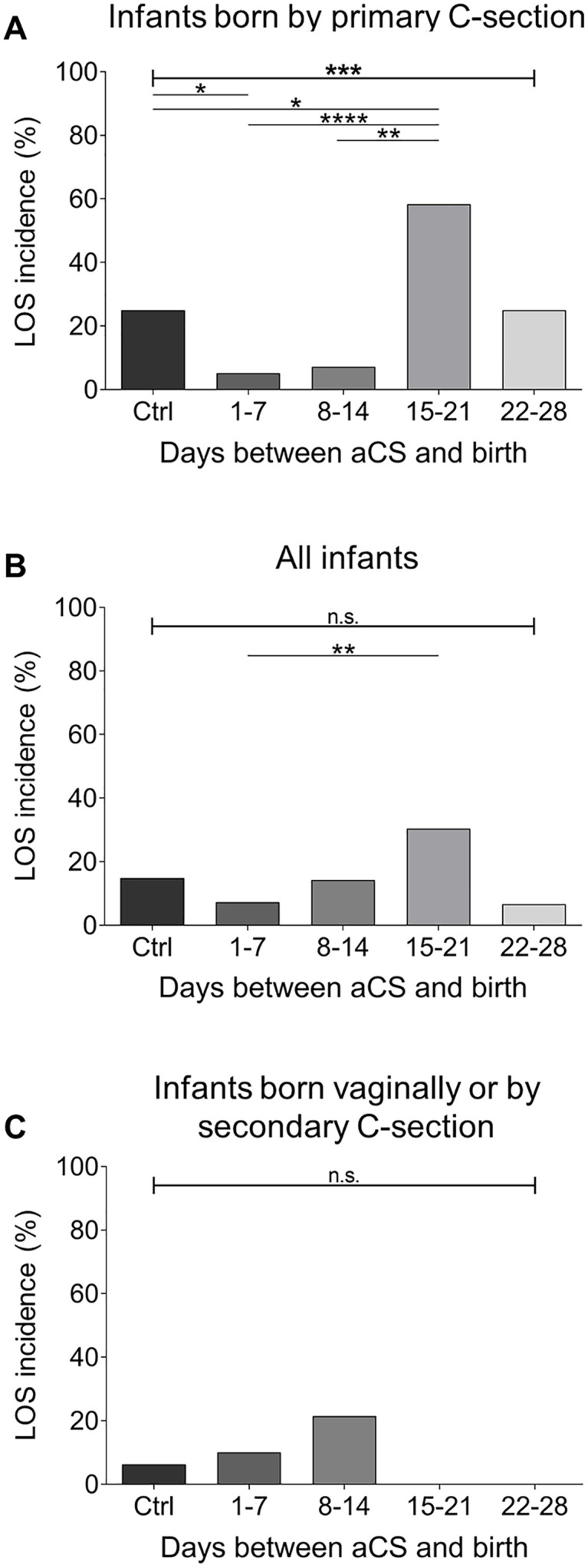
The incidence of LOS is influenced by aCS in dependence of the timing of administration before birth and the MOD. **A-C** Incidence of LOS in the subgroup of preterm infants delivered by primary C-section (*n* = 81) **(A)**, in the entire preterm infant cohort (*n* = 162) (**B**) and in the subgroup delivered either vaginally or by secondary C-section (*n* = 81) **(C)**, each stratified for the timing of aCS administration to the mothers in relation to birth. Eight (40%) cases of LOS were blood culture proven (one Escherichia coli, two Bacillus cereus, one CoNS, one Klebsiella pneumoniae, two Enterobacter aerogines, one Enterobacter cloacae). Differences were calculated using Chi^2^ tests, *p < 0.05, **p < 0.005, ***p < 0.001, ****p < 0.0001.

The incidence of LOS was significantly reduced among infants delivered by primary C-section when exposed to aCS within one week before birth compared to not aCS exposed control infants; in contrast, when the interval between aCS administration and birth exceeded 14 days, LOS incidence increased compared to the control group (Fig 1A). In contrast, when pooling all infants, the occurrence of LOS did not significantly change following aCS treatment compared with non–aCS-exposed control infants, regardless of the timing of aCS administration (Fig 1B). This absence of effect was also evident when the analysis was restricted to infants born with labor either vaginally or via secondary C-section, with no differences observed according to MOD within this subgroup (Fig 1C).

These data point to a differential impact of aCS on the risk of LOS in preterm infants in dependence of the timing of aCS and the MOD.

### aCS have opposing effects on postnatal S100A8/A9 serum levels in dependence of time interval to birth

**Fig 2:**
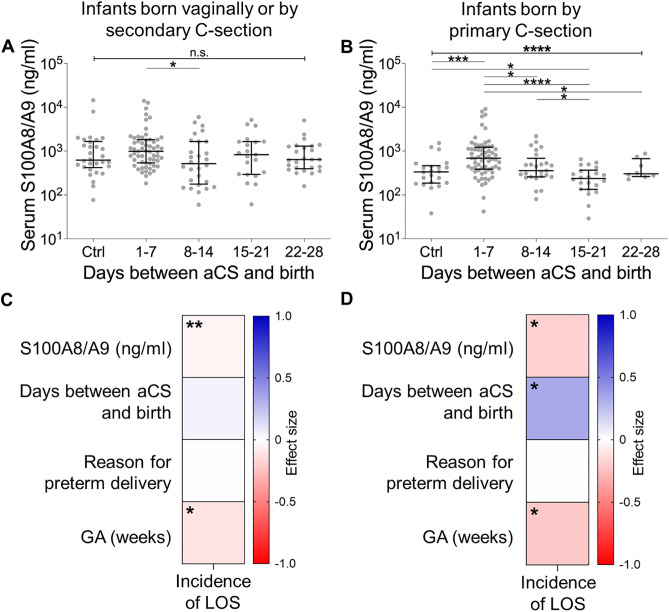
Postnatal S100A8/A9 serum levels in preterm infants depend on the timing of aCS administration before birth and correlate with the incidence of late-onset sepsis. **A, B** S100A8/A9 concentrations in serum samples (*n* = 141) obtained on day one to day three of life in preterm infants delivered vaginally or by secondary C-section (**A)** or by primary C-section **(B)**. Subgroups were stratified for the timing of aCS administration in relation to birth. Scatter plots show median and interquartile range. Data were analyzed using Kruskal-Wallis test with *post-hoc* Dunn’s multiple comparison test. **C, D** Correlations derived from a multiple logistic regression model including all indicated factors showing the effect direction and magnitude of day one to day three S100A8/A9 serum levels, timing of aCS administration, the reason for preterm delivery and the gestational age (GA) on the incidence of late-onset sepsis (LOS) in infants delivered vaginally or by secondary C-section (**C**) or by primary C-section **(D)**. Significances are indicated by asterisks. *p < 0.05, **p < 0.005, ****p < 0.0001.

We previously demonstrated that high postnatal S100A8/A9 serum levels are associated with a decreased risk of LOS in preterm infants [14]. To analyze whether the timing-dependent differential effects of aCS on the infants’ sepsis risk might be related to S100A8/A9 we determined the serum levels of S100A8/A9 during the first three days of life in the different aCS treatment groups. Within the group of infants delivered vaginally or by secondary C-section, no remarkable effect of aCS on S100A8/A9 serum levels was found (Fig 2A). This is most likely due to *a priori* equally higher S100A8/A9 serum levels following preterm labor and delivery via VD or secondary C-section, compared to infants delivered by primary C-section (S1 Fig and shown previously in another cohort [14]). Intriguingly, S100A8/A9 serum levels were higher following aCS administration within one week prior to birth compared to controls (Fig 2B) while lower S100A8/A9 serum levels were noted if the time interval between aCS administration and birth exceeded 14 days (Fig 2B). Multiple regression analyses revealed that the risk of LOS correlated with serum S100A8/A9 levels in all infants independent of the MOD whereas a correlation between the timing of aCS administration and the occurrence of LOS was only evident within the group of infants delivered by primary C-section (Fig 2C and 2D).

### Effect size of aCS on postnatal S100A8/A9 serum levels

**Fig 3:**
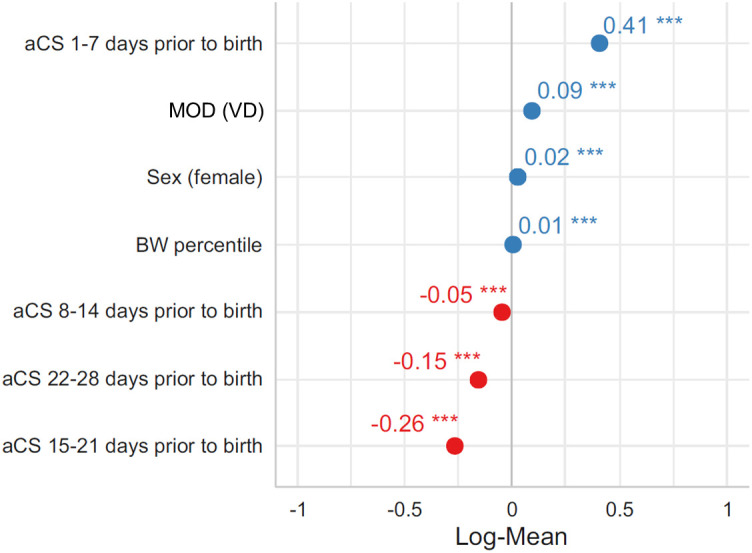
Among the main influencing factors aCS have the strongest impact on postnatal S100A8/A9 serum levels in preterm infants. Effect sizes building a generalized linear model of indicated S100A8/A9 influencing factors in the entire preterm infant cohort were plotted as log mean. All included factors showed a significant effect on postnatal S100A8/A9 serum levels with aCS administered within 7 days prior to birth showing the strongest increasing effect, whereas a robust decreasing effect was found when the interval between aCS administration and birth exceeded 7 days. ***p < 0.001.

In order to evaluate the effect size of aCS on S100A8/A9 serum levels in dependence of the timing of aCS administration we built a generalized linear model while accounting for factors such as MOD, BW percentile and sex previously identified to influence S100A8/A9 serum levels [14]. As expected, all considered factors showed a significant influence on the serum level of S100A8/A9 (Fig 3). Surprisingly, among these factors, the administration of aCS to the mother within a week from birth showed the strongest increasing effect. Moreover, a significantly decreasing effect on postnatal S100A8/A9 serum levels was found for aCS administration 8 or more days prior to birth. This effect was strongest if aCS had been administered 15–21 days before birth.

These findings show that aCS have a stronger impact on S100A8/A9 serum levels than hitherto known influencing factors with the effect direction being dependent on the timing of aCS administration.

### Nocturnal administration of aCS within one week prior to birth is associated with higher S100A8/A9 levels

**Fig 4:**
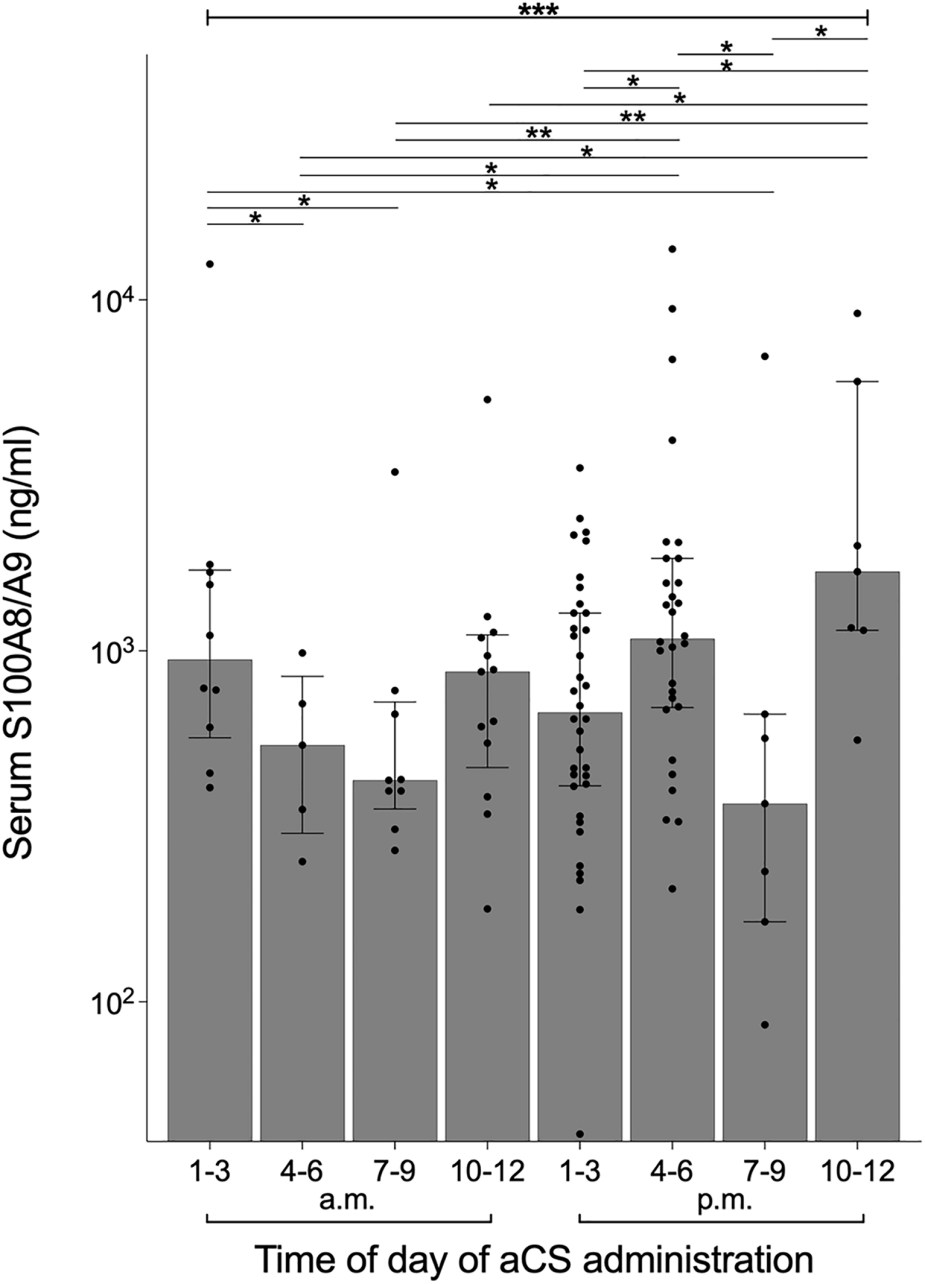
The increase of postnatal S100A8/A9 serum levels is strongest after nightly administration of aCS one week prior to birth. S100A8/A9 serum levels on the first three days of life in preterm infants whose mothers had received aCS 7 or less days prior to birth were grouped according to the time of day of aCS administration (*n* = 117 serum samples) showing an inverse pattern compared to the diurnal profile of corticosteroid levels in adults. Bars and whiskers show median and interquartile range. Data were analyzed using Kruskal-Wallis test and Mann Whitney *U* tests. *p < 0.05, **p < 0.01, ***p < 0.005.

As corticosteroid serum levels exhibit circadian rhythms, we conducted a secondary analysis to determine whether the timing of aCS administration during the day influenced its efficacy in increasing infant S100A8/A9 serum levels. This analysis was performed on the group of infants whose mothers had received aCS within one to 7 days prior to birth (n = 69). Postnatal serum levels of S100A8/A9 in neonates differed significantly depending on the time of day at which aCS was administered to the mother (Fig 4). The highest levels were observed after nighttime aCS administration, whereas the lowest levels occurred when aCS was given in the morning or evening. Notably, this pattern was opposite to the typical diurnal rhythm of corticosteroid levels in adults, which peak in the morning and evening and reach their lowest levels at night [27].

These data indicate that administering aCS at night – when endogenous steroid levels are physiologically low – is most effective in increasing S100A8/A9 serum levels in newborns.

## Discussion

The mechanisms underlying the benefit of aCS treatment regimens on respiratory outcomes are well acknowledged, while the impact of aCS on the risk of LOS still remains unclear. Our novel results suggest that aCS treatment increase postnatal S100A8/A9 serum levels in preterm infants resulting in a LOS-preventive effect as long as short time intervals between aCS and birth, i.e., < 8 days are obeyed.

Several previous studies could not detect a significant impact of aCS on the incidence of LOS in preterm infants [10–12], while a meta-analysis of 10 studies found a reduction of LOS after aCS treatment [1]. In our preterm infant cohort, we now revealed that a significant reduction in the incidence of LOS following aCS treatment relates only to infants born by primary C-section and whose mothers received aCS no longer than one week prior to birth. The dependence on the MOD and the timing of administration might account for the inconsistencies of previous reports on aCS effects on LOS.

The alarmin S100A8/A9 is massively released at birth, promotes a favorable and uneventful immune adaptation and protects from LOS by regulating innate immune responses [14–17,19]. While various studies in humans and mice reported already on an increased expression of S100A8/A9 in response to steroid treatment in disease conditions [20–22,28], this is to our best knowledge the first report on the impact of a corticosteroid treatment of mothers for imminent preterm delivery on S100A8/A9 serum levels in the preterm infant. In accordance with the above-mentioned studies [20–22,28], we found increased serum S100A8/A9 levels in preterm neonates whose mothers had received a full course of aCS within 7 days prior to birth. This effect was most significant in preterm infants delivered by primary C-section, which confirms our previous observation that S100A8/A9 serum levels are lower in infants born by C-section compared to vaginally delivered babies, particularly in those delivered by primary C-section at the absence of labor [14]. Noteworthy, this is the first report revealing that aCS had stronger effects on postnatal S100A8/A9 serum levels than hitherto identified factors influencing S100A8/A9 levels in preterm infants such as the MOD, BW percentile and sex [14].

However, and not examined in other steroid treatment studies, aCS administration had a decreasing effect on S100A8/A9 levels when more than 7 days elapsed between aCS treatment and birth. This imposes like a rebound phenomenon that decreases the endogenous production of corticosteroids as reported for corticosteroid treatments in other contexts [29,30], which in turn might cause the decrease of S100A8/A9 levels in the infant. However, such rebound phenomenon has rather been reported for higher doses and longer durations of steroid treatment and usually occurs earlier after treatment cessation [30,31]. Koenen et al. described a transient reduction in cortisol and adrenocorticotropic hormone (ACTH) levels in mothers after aCS treatment. Though, the nadir occurred already on day two after the first betamethasone dose [32] and levels recovered to about 50% of normal values within two days. Other studies even reported decreased serum cortisol and 17-hydroxyprogesteron levels on day one of life in preterm infants whose mothers had received aCS within 7 days prior to birth [33,34]. However, a comparison of serum cortisol levels on the first day of life between infants whose mothers had received aCS less or more than 7 days prior to birth showed no significant differences [35]. Thus, the important observation of significantly decreased S100A8/A9 serum levels in preterm infants of mothers who received aCS 14 and more days prior to delivery seems not simply to be due to a corticosteroid rebound phenomenon and needs further clarification in future studies.

The timing-dependent differential effects of aCS on LOS and postnatal S100A8/A9 serum levels correlated with each other in a strictly inverse manner corroborating that the aCS effect on LOS is mediated by S100A8/A9 [14–17]. The primarily low S100A8/A9 serum levels in infants born by primary C-section explain well why these infants benefit most from aCS within one week prior to birth in terms of prevention of LOS. High postnatal S100A8/A9 serum levels represent an age-specific, essential mechanism of immune regulation that protects neonates from hyperinflammatory immune responses and allows a favorable course of immune adaptation and microbial colonization after birth [14–17,19]. From a clinical perspective, the modulation of S100A8/A9 holds potential as a potent protective measure against sepsis in neonates, especially in preterm infants, who are at the highest risk of sepsis.

Previous studies provided evidence for an influence of the daytime of aCS administration in relation to the circadian rhythm on infant morbidity. Neonatal hypoglycemia occurred more often after a morning administration compared to an administration at night [36]. In a human observational study, aCS injections out of phase of the diurnal rhythm were associated with an increased susceptibility to behavioral impairment in 5-year-old preterm infants [37]. In our study cohort, the strongest increase of postnatal S100A8/A9 levels was evident after aCS administration at night when endogenous corticosteroid levels are physiologically lowest [27]. However, in many cases the time of day for aCS administration cannot be chosen electively as treatment needs to be started as soon as possible in the face of imminent preterm birth. In our cohort, 43 percent of women gave birth in the optimal interval window of 7 days after aCS treatment, which is in line with previous studies [8,38], whereof only 19 percent received the aCS treatment at night.

Our study has limitations, which are inherent to single center design and convenience sample recruitment, which especially in subgroup analyses resulted in smaller sample sizes limiting strength of significances. We adjusted for important risk factors of LOS, however, cannot account for all clinical factors related to time the interval between aCS and decision to deliver a preterm infant which remains at the discretion of the attending obstetrician. This may introduce residual confounding despite comprehensive regression adjustments. The observational design of the study only allows the identification of associations, but not the demonstration of the underlying mechanistic relationships, which requires further research.

## Conclusion

Our data provide evidence that aCS impacts on postnatal S100A8/A9 serum levels and reduces the risk of LOS in preterm infants born by primary C-section. The effect of aCS is dependent on the timing of administration. It increases S100A8/A9 serum levels and minimizes the risk of LOS, if given one week prior to birth, but has opposite effects if this interval exceeds more than two weeks. It works best if applied at night with respect to the risk reduction of LOS in infants delivered in the absence of labor.

Our findings, derived from a prospective birth cohort of preterm infants, provide robust evidence that aCS administration not only affects neonatal outcomes but that its timing relative to birth and time of day critically influences postnatal immune status and the risk of LOS. Importantly, the prospective nature of the cohort strengthens the validity of the observed associations, while still warranting cautious interpretation with respect to causality.

While current standards of care focus primarily on the indication for and type of aCS administration, our data suggest that optimization of administration timing—particularly in preterm infants delivered by primary C-section in the absence of labor—may substantially enhance clinical benefit. Furthermore, our study might have future clinical implications on S100A8/A9 supplementation strategies. However, before these findings can be translated into a revised standard of care, confirmatory studies, ideally multicenter and interventional in design, are needed to validate the observed timing-dependent effects and to assess generalizability across different clinical settings.

Future research should further elucidate the mechanistic link between circadian steroid signaling and induction of S100A8/A9. In parallel, long-term follow-up studies are required to evaluate the safety and developmental consequences of refined aCS timing strategies.

Together, these data provide a compelling rationale for exploring a more individualized, time-aware approach to aCS administration in preterm birth without labor. If validated, such an approach could inform future clinical guidelines and contribute to improved prevention of LOS in this highly vulnerable population.

## Supporting information

S1 Table Clinical and laboratory signs of infants diagnosed with LOS with or without positive blood culture. Number of positive clinical or laboratory signs did not differ between infants with and without positive blood culture (p = 0.7501 and p = 0.6725). Data were analysed using Mann Whitney *U* tests.
(DOCX)

S1 Fig Postnatal S100A8/A9 serum levels in preterm infants depend on the MOD. S100A8/A9 concentrations in serum samples obtained on day one to day three of life in the control group in dependence of the MOD (*n* = 52). Scatter plots show median and interquartile range. Data were analyzed using Mann Whitney *U* test. ***p < 0.001. VD, vaginal delivery.
(TIF)

## Abbreviations

aCS: antenatal corticosteroids
BW: birth weight
CoNS: coagulase negative staphylococci
GA: gestational age
LOS: late-onset sepsis
MOD: mode of delivery
