# Supplementary material for: S100-alarmins, antenatal corticosteroids and the risk of late-onset sepsis in preterm infants: A prospective cohort study
Source: PLoS One. 2026 Jan 27;21(1):e0341544. doi: 10.1371/journal.pone.0341544 (PMC12843532; doi:10.1371/journal.pone.0341544)
Supplement: S1 Table — Number of positive clinical or laboratory signs did not differ between infants with and without positive blood culture (p = 0.7501 and p = 0.6725). Data were analysed using Mann Whitney U tests. (DOCX) [file pone.0341544.s001.docx]

**S1 Table. Clinical and laboratory signs of infants diagnosed with LOS with or without positive blood culture.** Number of positive clinical or laboratory signs did not differ between infants with and without positive blood culture (p=0.7501 and p=0.6725). Data were analysed using Mann Whitney *U* tests.
