# Supplementary figures and images for: S100-alarmins, antenatal corticosteroids and the risk of late-onset sepsis in preterm infants: A prospective cohort study
Source: PLoS One. 2026 Jan 27;21(1):e0341544. doi: 10.1371/journal.pone.0341544 (PMC12843532; doi:10.1371/journal.pone.0341544)

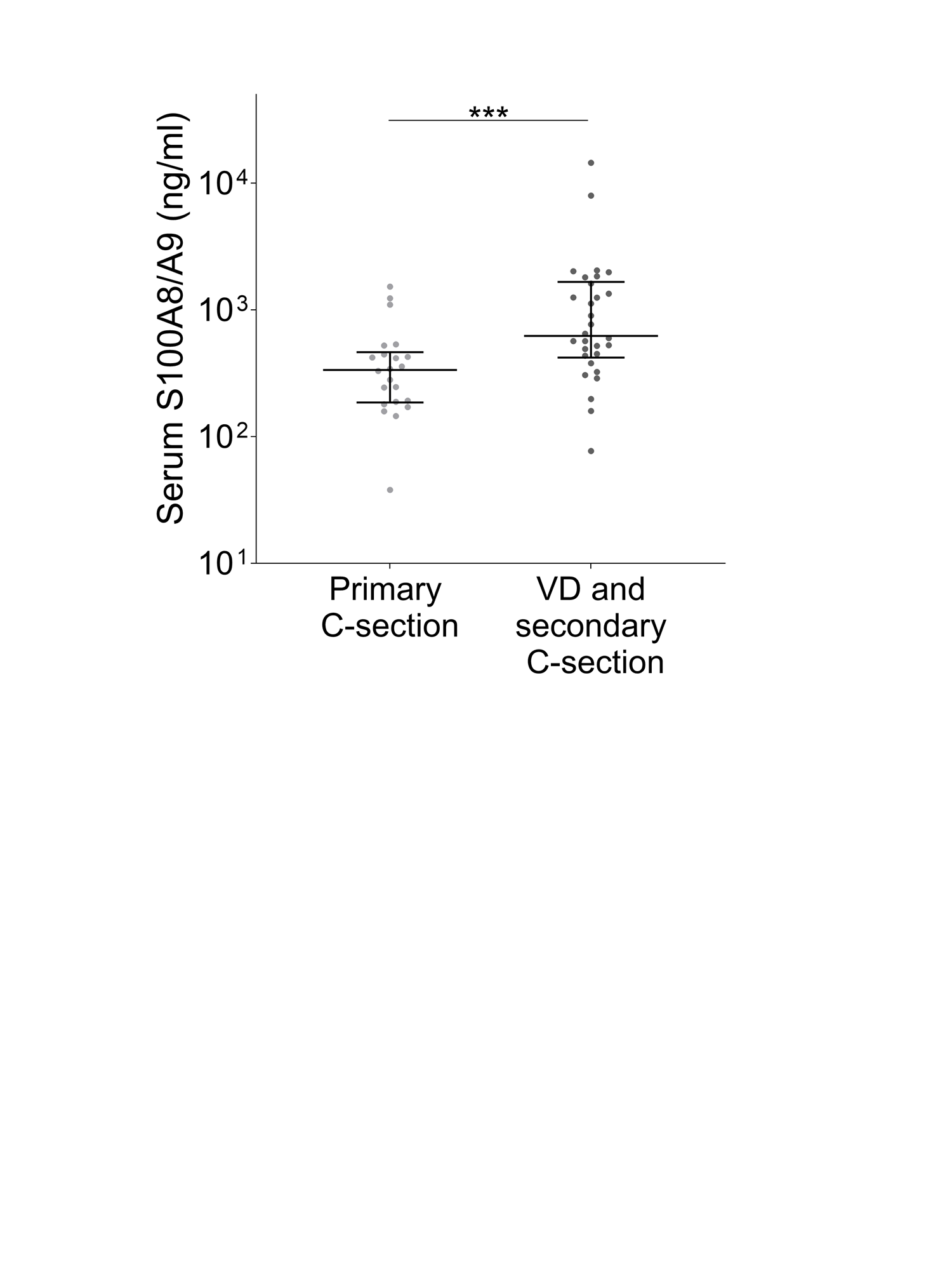

Supplement: S1 Fig — S100A8/A9 concentrations in serum samples obtained on day one to day three of life in the control group in dependence of the MOD (n = 52). Scatter plots show median and interquartile range. Data were analyzed using Mann Whitney U test. ***p < 0.001. VD, vaginal delivery. (TIF) [file pone.0341544.s002.tif]
